# Editorial: Advancements in the understanding of Mucorales biology and the management of mucormycosis

**DOI:** 10.3389/fcimb.2024.1427252

**Published:** 2024-05-21

**Authors:** Georgios Chamilos, Victoriano Garre

**Affiliations:** ^1^ Laboratory of Clinical Microbiology and Microbial Pathogenesis, School of Medicine, University of Crete, Heraklion, Crete, Greece; ^2^ Institute of Molecular Biology and Biotechnology, Foundation for Research and Technology, Heraklion, Crete, Greece; ^3^ Departamento de Genética y Microbiología, Facultad de Biología, Universidad de Murcia, Murcia, Spain

**Keywords:** mucormycosis, animal models, diagnosis, invasion, host response, COVID-19-associated mucormycosis, treatment

Mucormycosis is a life-threatening infection caused by certain species of the fungal order Mucorales. Although respiratory tract is the primary site of infection by Mucorales, mucormycosis can affect different organ and manifesting as rhino cerebral, pulmonary, cutaneous, gastrointestinal, and disseminated disease (Alqarihi et al., 2023). This opportunistic infection affects patients with defects in the numbers and/or function of professional phagocytes and also individuals with unique metabolic defects. In particular, poorly controlled diabetes mellitus, acidosis, malnutrition, acquired iron overload syndromes, trauma or burns, especially in the setting of natural disasters (e.g., tornados, tsunamis), are unique predisposing conditions for mucormycosis. While the exact global burden of mucormycosis remains unknown, its incidence is steadily increasing over the past three decades. The COVID-19 pandemic has dramatically increased this trend, resulting in a surge of up to 50 times the previously recorded maximum cases (Hoenigl et al., 2022). Alarmingly, the mortality rates of mucormycosis exceed 40% and approach almost 100% in disseminated type of the disease. This high mortality rate, which has shown little improvement in recent years, is attributed to the delay in diagnosis, the limited therapeutic options, and the incompletely understood immunopathogenesis of the disease. In particular, necrosis and angioinvasion are prominent pathogenic features of mucormycosis that account for rapid dissemination and poor response to antifungals. Additionally, the intrinsic antifungal resistance of Mucorales to most available antifungal drugs is poorly characterized at the molecular level (Lax et al., 2024). A better understanding of Mucorales biology, including the molecular mechanisms governing physiological immune response, pathogenesis, and antifungal resistance, may lead to improved management of the disease. Through this Research Topic, we aim to provide the most recent advances in understanding the development and treatment of the infection.

Prompt diagnosis and early initiation of amphotericin B based therapy are crucial in reducing mortality rates (Chamilos et al., 2008). As opposite to other invasive mold infections, there are no available diagnostic biomarkers for mucormycosis. The review by Alqahiri et al. in this Research Topic offered an updated overview of current available procedures of diagnosis, highlighting the challenges associated with traditional methods, many of which exhibit reduced sensitivity and specificity. However, recent advancements in non-invasive molecular diagnostics, particularly polymerase chain reaction (PCR)-based technologies, hold promise for expediting diagnosis. Real-time PCR (qPCR), primarily based on detecting ITS1 or ITS2 ribosomal regions or the 18S region of rDNA, emerges as a promising tool for early mucormycosis diagnosis. Multicenter studies have demonstrated good reproducibility, sensitivity, and specificity in serum samples from patients with different manifestations of mucormycosis, suggesting transfer of Mucorales DNA into the bloodstream (Rocchi et al., 2021; Millon et al., 2022). Detection of Mucorales cell-free DNA in serum, facilitated by the extensive angioinvasion of the infection, can aid in early diagnosis, targeted antifungal treatment, and disease management by monitoring Mucorales DNA load (Millon et al., 2022). The promising potential of qPCR for mucormycosis diagnosis has led to the development of at least three commercial qPCR kits for Mucorales, currently under evaluation for testing in serum, bronchoalveolar lavage (BAL), or biopsies, with initial data indicating promising sensitivity and specificity (Lax et al., 2024). Despite the excellent potential of qPCR in serum for early diagnosis of mucormycosis, additional refinements of the technique are necessary to translate it into routine clinical practice for patients with possible fungal infection.

The study of Thornton et al., in the present Research Topic followed a similar approach of detecting Mucorales biomarkers in human serum and BAL, utilizing a pan-Mucorales test based on competitive lateral-flow antigen detection device that allows rapid and specific detection of most Mucorales species causing mucormycosis. This assay uses an IgG2b monoclonal antibody (mAb) that binds to extracellular polysaccharide (EPS) antigens secreted during hyphal growth of Mucorales fungi. The specificity is ensured by the absence of mAb cross-reactivity with other clinically importance fungi, including *Aspergillus*, *Candida*, *Cryptococcus*, and *Fusarium* species. The test successfully detected Mucorales EPS in human serum and BAL, with limit of detection in human serum within the sensitivity range for competitive lateral-flow immunoassays (Di Nardo et al., 2021). The simplicity, speed, and affordability of this assay make it a potentially valuable tool for point-of-care diagnosis, enabling early detection of mucormycosis, particularly in low- to middle-income countries. Further investigations are warranted to assess the relevance of this assay across different manifestations of mucormycosis, providing an opportunity for clinical evaluation.

The treatment of mucormycosis is hindered by limited knowledge of disease development. Reliable models to study the disease are therefore essential. While mammalian animals are physiologically relevant models, ethical and economic considerations restrict their use, which should adhere to the 3R policy of replacement, reduction, and refinement in animal research (Tannenbaum and Bennett, 2015). In this Research Topic, Scheler and Binder updated the information on alternative animal models available, ranging from insects to zebrafish. Special emphasis was placed on novel alternative model animals or adapted methods established in the last three to four years, discussing advantages and limitations of each model. Their use is particularly crucial in high-throughput screenings requiring a large number of individuals to understand pathogenicity factors and evaluate novel treatment regimens. However, these models are currently only available for disseminated infections, and there is a lack of standardized procedures and protocols. Despite these limitations, their use is fostering our understanding of previously undescribed virulence factors and unveiling pathogen recognition and immune evasion strategies.

The use of animal models and mammalian cell cultures has advanced our knowledge about disease pathogenesis. Alqihiri et al., in the present Research Topic, reviewed pathogenesis, along with other aspects of the disease. Tremendous strides have been made in understanding Mucorales tissue adhesion and invasion, host response, and pathogenicity factors. The implementation of new techniques to manipulate the genomes of mucormycosis-causing species is expected to increase in the coming years, particularly following the successful use of CRISPR/Cas9 system in *Rhizopus* species (Lax et al., 2021), which should pave the way for further disease characterization. Alqihiri et al. also described current available treatments, as well as those in development, including those based on the latest acquired knowledge, such as antibodies against pathogenicity factors and the use of adjunctive therapies. Regarding the improvement of treatment with current antifungals, Liu and Ma described in the present Research Topic the remarkable efficacy and safety of treating patients with mucormycosis complicated by hematological malignancies with amphotericin B colloidal dispersion. This treatment showed remarkable efficacy and safety, as none of the patients suffered from renal injury. However, due to the limitations such as the study being conducted at a single center and small sample size, larger-scale, multicenter studies are required to confirm the results.

In conclusion, the current Research Topic has contributed to improve our knowledge of promising new diagnostic methods for early detection of Mucorales, the optimal treatment of patients with amphotericin B colloidal dispersion, the use of alternative animal models, and the latest advances in the pathogenesis, epidemiology, virulence factors, diagnosis, and treatments. Despite major advances in fungal biology research, it is urgent to involve more researchers and medical doctors in the study of mucormycosis and Mucorales biology to develop more effective diagnosis tests and treatments. The growing availability of sequenced genomes and molecular tools is expected to play an important role in increasing the interest for the disease. The generation of biobanks of clinical samples from patients with mucormycosis is an unmet need for translational studies on immunology, pathogenesis, and diagnostics of this devastating disease. The recent development of novel technologies that allow for spatial high throughput gene expression analysis and protein profiling at the single cell level in paraffin embedded tissue biopsies, will further advance our understanding of the immunopathogenesis of mucormycosis in humans. In view of these challenges and opportunities, international efforts to create collaborative research networks including experts in diverse fields of Mucorales research are a timely initiative.

## Author contributions

VG: Conceptualization, Writing – original draft, Writing – review & editing. GC: Conceptualization, Writing – original draft, Writing – review & editing.
